# OMNIVIL—An Autonomous Mobile Manipulator for Flexible Production

**DOI:** 10.3390/s20247249

**Published:** 2020-12-17

**Authors:** Heiko Engemann, Shengzhi Du, Stephan Kallweit, Patrick Cönen, Harshal Dawar

**Affiliations:** 1 IaAM Institute, Faculty of Mechanical Engineering and Mechatronics, University of Applied Sciences Aachen, 52074 Aachen, Germany; kallweit@fh-aachen.de (S.K.); coenen@fh-aachen.de (P.C.); dawar@fh-aachen.de (H.D.); 2 Faculty of Engineering and Built Environment, Tshwane University of Technology, Pretoria 0001, South Africa

**Keywords:** autonomous mobile manipulation, workspace monitoring, flexible manufacturing, intelligent robots

## Abstract

Flexible production is a key element in modern industrial manufacturing. Autonomous mobile manipulators can be used to execute various tasks: from logistics, to pick and place, or handling. Therefore, autonomous robotic systems can even increase the flexibility of existing production environments. However, the application of robotic systems is challenging due to their complexity and safety concerns. This paper addresses the design and implementation of the autonomous mobile manipulator OMNIVIL. A holonomic kinematic design provides high maneuverability and the implemented sensor setup with the underlying localization strategies are robust against typical static and dynamic uncertainties in industrial environments. For a safe and efficient human–robot collaboration (HRC), a novel workspace monitoring system (WMS) is developed to detect human co-workers and other objects in the workspace. The multilayer sensor setup and the parallel data analyzing capability provide superior accuracy and reliability. An intuitive zone-based navigation concept is implemented, based on the workspace monitoring system. Preventive behaviors are predefined for a conflict-free interaction with human co-workers. A workspace analyzing tool is implemented for adaptive manipulation, which significantly simplifies the determination of suitable platform positions for a manipulation task.

## 1. Introduction

In the last decades, the process of automated manufacturing was designed for constant large production batches and well-defined product types. This kind of production is typically supported by static industrial robots, which offer a high repeatability of periodic tasks. In recent years, industrial automation has changed. The huge interest in customized products generates the need for flexible automation [1]. This individual production of user configurable goods, ends up in numerous product variations and rapid response to customer requirements through short-cycle product substitutions [2]. Typical advantages of automated assembly lines like high throughput rates and high repeatability, which result in product cost reduction, are reduced by necessary adjustments of the production process. Manual production would address the novel needs in terms of flexibility and agility [3]. A complete manual production is out of question in most cases, due to the lack of repeatability, the low cost-efficiency and the complex design of today’s goods. The greatest challenge of nowadays manufacturing industry is to improve the flexibility and agility of the automated manufacturing processes.

One approach is the implementation of "mobile industrial manipulators" in the production process. The term mobile industrial manipulator describes an industrial robot arm (manipulator) mounted on an autonomous mobile robot (AMR). A mobile industrial manipulator offers more flexibility and agility regarding its workspace, which is extended by the AMR’s mobility. Common industrial navigation concepts are based on line follower procedures [4] or passive [5] and active [6] landmark detection. These methods are approved in industrial environments, but not as flexible as desired. In research on the other hand, the advanced concepts of mobile robotics, like perception and navigation, are used to improve the mobility for autonomous applications. These "autonomous industrial mobile manipulators" (AIMM) [7] are capable of autonomous navigation, even in dynamic environments. Furthermore, the perceived data can be used to realize a shared human–robot–workspace.

**Figure 1:**
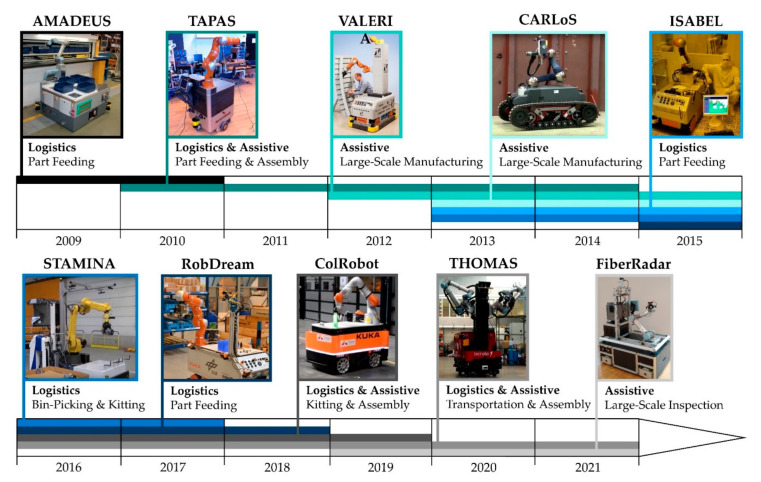
Related research projects from 2009 to 2021.

One of the first AIMMs was MORO [8], which was introduced in 1984. MORO is capable of navigating on the shopfloor and executing pick and place tasks. Starting from this pioneer work a lot of further developments were carried out in the related research fields. Figure 1 shows an overview of related research projects conducted over the last decade. In addition, it includes the basic field of application and the used mobile industrial manipulator.

In the research project TAPAS [9] the AIMM Little Helper [10] was developed. Little Helper shows the potential of the technological concept for logistic and assistive tasks in industrial environments. Therefore, scenarios of industrial applications were reproduced in experimental environments for multipart feeding [11] and multirobot assembly [12]. A comparable concept was followed by the project AMADEUS [13]. In an industrial example scenario, the developed AIMM Amadeus successfully refilled production parts at a manual workbench. The project ISABEL [14] addressed transport and handling tasks in semiconductor production and life science laboratories. The research focus was set on perception and motion planning using GPU-based voxel collision detection [15,16]. Kitting and Bin-Picking scenarios were studied in the research project STAMINA [17]. The developed skill-based system SkiROS [18] is integrated in a manufacturing execution system (MES). In contrast to classical approaches, the world model, which represents the environment of the AIMM, will adapt itself to changes based on sensor data. As a result, the AIMM is able to operate in an unknown environment and to manipulate unknown objects without prior knowledge [19]. The research projects VALERIA [20] and CARLoS [21] addressed large-space manufacturing applications. The developed AIMM VALERIA is specifically designed for a collaboration between the human co-worker and the AIMM [22,23]. Visual workspace monitoring and a tactile skin secure a safe interaction [24]. In the project CARLoS, an AIMM was developed for automatic stud welding inside a ship hull. It features an intuitive human–robot interaction (HRI) by implementing a cursor based interface [25]. A new concept of a dreaming robot is introduced in the project RobDream [26]. The basic idea is to improve the capabilities of an AIMM by analyzing data in inactive phases. In an industrial example scenario the AIMM was used for production part delivery services [27]. The research project ColRobot [28] continues some aspects of the VALERIA project. It includes two industrial case studies: Trim part assembly in a van and the preparation and delivery of assembly kits for satellite assembly. The research contribution covers different fields such as object recognition [29], cartesian positioning of manipulators [30] and precision hand-guiding of the end-effector [31]. In the research project THOMAS [32] an AIMM [33] is a core element to create a dynamically re-configurable shop floor [34]. The AIMM should be able to perceive its environment and to cooperate with other robots and human co-workers [35]. A large-scale inspection task is carried out in the project FiberRadar [36]. The development addresses the monitoring of the wind turbine blade production process.

In this paper, we present OMNIVIL, an autonomous mobile manipulator. We provide a holistic overview from the design to the implementation of OMNIVIL in a model factory. The first step includes the novel design and construction of the mobile platform based on an omnidirectional kinematic and standard industrial component. A collaborative manipulator is mounted on top of the platform. The control system is split into lower-level and higher-level tasks, whereby the implemented interface uses the standard Open Platform Communications Unified Architecture [37] (OPC UA). Real-time critical tasks are executed and managed on a programmable logic controller (PLC) with real-time capabilities. For example, the execution of motion commands or the monitoring of the hard real-time safety components. Tasks with a high computational load, like autonomous navigation, workspace monitoring and 6D motion planning are executed on a high-level system, which is an industrial PC.

The following strategies are implemented towards safe and efficient human–robot collaboration (HRC): (1) a novel workspace monitoring concept is presented to address the safety issue when implementing an AIMM, using RGB and thermal images as well as Lidar data; (2) the multilayer sensor setup is improved by the implementation of redundant algorithms for human co-worker detection based on neural networks. The resulting confidence intervals in 3D space are fused by applying the logarithmic opinion pool; (3) the presented navigation and manipulation concepts are developed to simplify the implementation of mobile manipulators in general. The developed zone-based navigation concept can easily be adapted to different production layouts and provides the functionality to switch between different motion behaviors; (4) dynamic zones, based on the 2D position of human co-workers provided by the workspace monitoring system, enable a controlled HRC; and (5) a visualization of the reachability and manipulability of the manipulator simplifies the process of identifying feasible handling spots for a workstation. The visual-servoing approach uses landmarks, detected by an RGB camera.

The rest of the paper is structured as follows: Section 2 describes the design of the mobile manipulator and the implemented sensor concept. Section 3 presents the control system, including the novel workspace monitoring system and the navigation concept. In Section 4, two experiments are demonstrated to evaluate and discuss the navigation capability of the mobile platform and the performance of the workspace monitoring system. Section 5 concludes the paper.

### Safety and Complexity in the Domain of AIMMs

The discussed research projects demonstrate the general application potential of AIMMs. A detailed study [38] analyzed 566 manual manufacturing tasks in five different factory types. Approximately 71% of the tasks were determined solvable using an AIMM. However, the technology has not found its way into industry yet [39]. The main reasons are safety concerns and the high complexity of an AIMM.

In an industrial context, the concept of HRC can lead to more flexibility and cost reduction, especially when the production layout is changed often [40]. The level of collaboration can be classified into the four different categories: coexistence, interaction, cooperation and true collaboration [41]. Except for the basic level of coexistence, every level includes a shared human–robot workspace. Therefore, safety regulations are the main technical issues to enable HRC [42]. The use of power and force limited lightweight manipulators has a high potential to widely enable the integration of AIMMs in the manufacturing industry. These types of manipulators are often called collaborative manipulators. In addition, new approaches combine hard real-time safety components (hard-safety), like verified industrial-grade sensors [22], with soft real-time safety components (soft-safety) such as tactile sensors [23,24] or vision based object detection [43]. However, preventing physical harm is only the first step to setup a stress-free and intuitive collaboration [44]. The cognitive or mental workload characterize how beneficial a collaboration between a human and a robot is [45]. The situational adaptation of the robot’s behavior reduces the mental workload of the human co-worker and simultaneously increases the productivity of the AIMM. Therefore, the perception of the environment in a human like manner is a key requirement. Classical approaches, which rely on safety zones or pure obstacle detection, do not provide sufficient meaningful information.

Following [46], an AIMM can be defined as a complex system, as it provides all of the following characteristics:

- It contains a collection of many interacting objects (software-modules and hardware-components).
- It makes use of memory or feedbacks.
- It can adapt its strategies based on stored data or feedback.
- It is influenced by its environment.
- It can adapt to its environment.
- The relations between the objects and the relation between the system and the environment is non-linear and non-trivial.

Following this definition, the complexity of AIMMs has significantly increased over the last decade. The aim of this development is to enable AIMMs to fulfill their tasks without human supervision or instructions. As a result, AIMMs have proven that they are able to autonomously perform various tasks: from logistics, to pick and place or complex handling. However, the implementation of AIMMs in production processes still requires human instructors or operators. Typical examples are the definition of navigation routes or park positions at a workstation as well as the teaching of manipulator motions. Reducing the complexity in this subarea would make this technology more feasible for widespread industrial use. In science many approaches exist to measure the level of complexity. One approach is to measure the time and resources needed to execute a task [47]. This concept can as well be applied to the implementation process of AIMMs. In the industrial context, the time required and the knowledge base needed by the human instructor are comparable quantities.

## 2. Robot Design

### 2.1. Mobile Platform

The positioning accuracy and the maneuverability are the major concerns of the mobile platform development. A holonomic kinematic is a special case of omnidirectional kinematic, which enables the platform to move in all directions without changing its orientation prior to moving. Therefore, holonomic kinematics are not subject to any kinematic restrictions, which means all movement commands can be executed instantly.

In this paper, the designed mobile platform is based on four Mecanum wheels [48] providing a holonomic kinematic behavior. Mecanum wheels are susceptible to ground unevenness, which will cause slip and other unexpected motion behaviors. A dedicated wheel suspension must ensure continues ground contact for each individual wheel. A rigid suspension of four wheels is a statically indefinite system, where continues ground contact is not guaranteed. A vertical spring-loaded suspension for each individual wheel solves this problem [49]. However, such a suspension will lead to inaccurate positioning of the manipulator during motion. Usually, the floor in industrial production environments is almost flat. Under this assumption, a pivoting axle for two of the four wheels will result in a statically determined system. The displacement of the wheel support points during a pendulum movement is minimized by centering the pivot point of the pendulum axle in the radial axle of the wheels.

**Figure 2:**
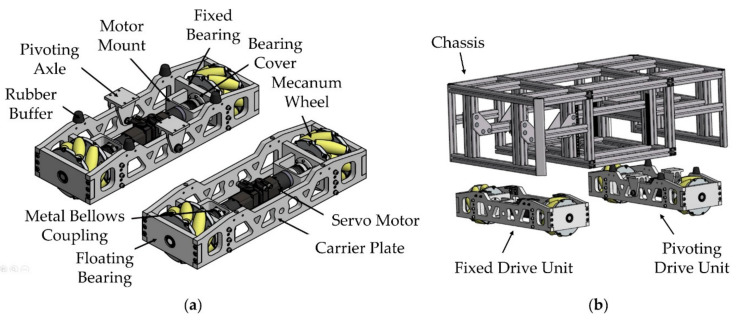
Mechanical components of the mobile platform. (**a**) Drive units; (**b**) drive units positioned at chassis.

Figure 2 shows the developed drive units and the chassis made from aluminum profiles. The drive units use a fixed/floating bearing for each wheel. Metal bellow couplings secure the servo drives against unexpected forces during motion. The pivoting axle is supported by rubber buffers to avoid direct contact between the pivoting drive unit and the chassis in extreme situations.

The general technical specifications of the developed mobile platform are listed in Table 1.

**Figure 3:**
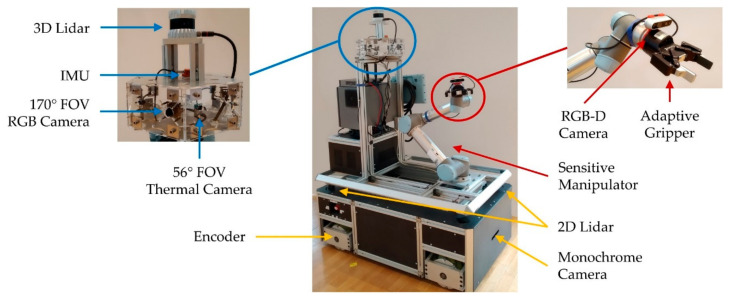
Sensor setup of the mobile manipulator OMNIVIL.

The collaborative manipulator UR5 is mounted on top of the mobile platform as shown in Figure 3. The manipulator is equipped with an adaptive gripper 2F-85 from ROBOTIQ. The adjustable gripping force and the detection of a successful grasp process makes this gripper suitable for collaborative robots.

### 2.2. Sensor Concept

The mobile manipulator is equipped with the following sensors to perceive its environment, as shown in Figure 3:

- two 2D Lidar (Sick TIM-S),
- one 3D Lidar (ouster OS0-32),
- one RGB-D camera (Intel-RealSense 435),
- three RGB cameras (ELP USBFHD04H-L170),
- one monochrome camera (UI-3251LE),
- six thermal cameras (Flir Lepton),
- one inertial measurement unit (IMU) (Xsens MTi-10) and
- four encoders.

The RGB and thermal cameras as well as the 3D Lidar are part of a multilayer workspace monitoring system to detect human co-workers or other potential collision objects. The cameras are mounted in a hexagon. The thermal camera provides a horizontal field of view (FOV) of 56°. The RGB camera provides a 170° FOV. The 2D Lidars, the IMU, the encoders and the monochrome camera are used for the autonomous navigation of the platform. In addition, the 2D Lidars are part of the lower-level emergency stop circuit. The data provided by the attached RGB-D camera is used to calculate grasp positions and to avoid collisions during the motion of the manipulator.

## 3. Control System

### 3.1. Components and Connections

The mobile platform is equipped with a PLC. The B&R PLC-X20 is connected via Powerlink to two APOCOSmicro servo drives. Each servo drive controls two servo motors. The battery module consists of a lithium iron phosphate (LiFePo4) accumulator and an integrated battery management system (BMS). It provides a nominal capacity of 50 Ah at a nominal voltage level of 48 V.

**Figure 4:**
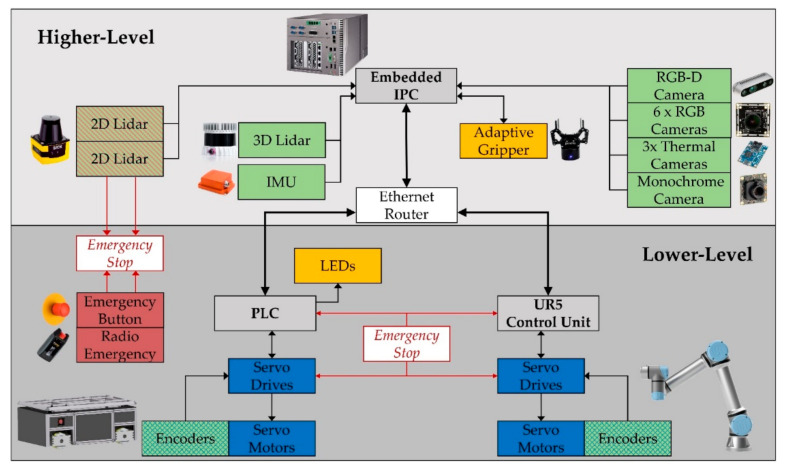
Components and connections of the control system: sensors (**green**), actuators (**blue**), controllers (**gray**), emergency (**red**), and additional peripheral devices (**orange**).

Figure 4 shows a schematic overview of the components and the interior connections of the mobile manipulator. Both lower-level controllers and servo drives are connected to an emergency stop circuit to provide functional safety. An emergency stop can be triggered by an emergency button located at the mobile platform, through a Tyro radio emergency button or by the 2D safety Lidars.

The higher-level controller is an embedded industrial computer (Vecow GPC-1000). The embedded computer is connected to the PLC and the UR5 control unit via a gigabit ethernet router. The router can be integrated in any production environment specific Wi-Fi infrastructure as an access point.

### 3.2. Software Architecture

The architecture of the control software is also divided into lower- and higher-level tasks. The higher-level software modules are based on the Robot Operating System (ROS) [50]. The developed modules provide the following functionalities:

- three-dimensional 360° workspace monitoring,
- autonomous navigation in unstructured dynamic environments,
- 6 Degree of Freedom (DoF) adaptive manipulation and
- task management in form of a state machine.

**Figure 5:**
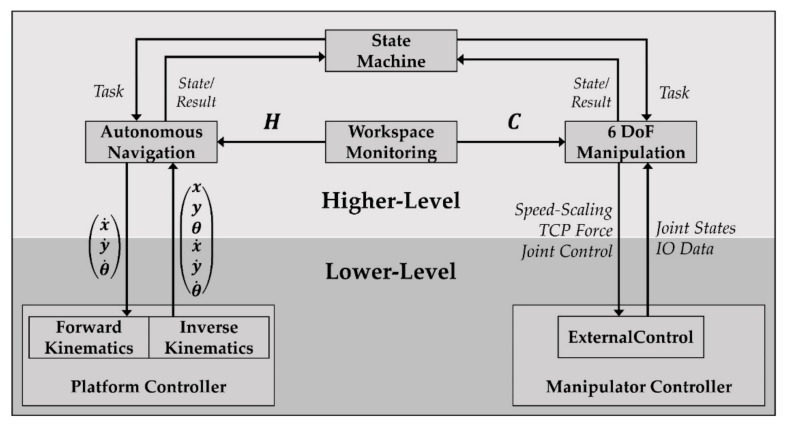
Main modules of the control software.

The lower-level control executes real-time critical tasks and commands provided by the higher-level control software. The platform controller makes use of a standard kinematic model as defined in [51]. Figure 5 shows a schematic overview of the main software modules of the higher-level and their interaction with the lower-level.

The integrated state machine manages the global task execution including navigation and manipulation tasks. The multilayer workspace monitoring system determines the position of human co-workers in form of a two-dimensional heatmap H, which is used for autonomous navigation. In addition, it provides a point cloud C to the 6 DoF manipulation module for collision avoidance based on pure obstacle detection.

### 3.3. Workspace Monitoring System

The mobile manipulator shares the workspace with human co-workers. Therefore, the lower-level hard-safety concept is extended by an additional higher-level workspace monitoring system (WMS). The WMS detects human co-workers and estimates their 2D position in the robot coordinate system R. This approach enables the robot to preventively react to humans in its workspace, e.g., reduce its velocity, plan an alternative path or even stop, before the hard-safety is triggered. The intended concept enables a more intuitive cooperation between the human co-worker and the mobile manipulator.

**Figure 6:**
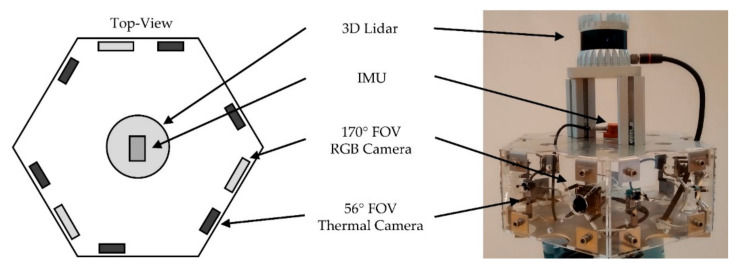
Workspace monitoring system in hexagon form.

The WMS covers a 360° horizontal FOV. Its reliability is increased through a multilayer sensor configuration. Therefore, the sensors are mounted in a hexagon (see Figure 6). The integrated 3D Lidar provides an ultra-wide 90° vertical FOV and a vertical resolution of 32 channels, which makes it suitable for 3D obstacle detection even in close distance to the mobile manipulator.

The process of determining the positions of human co-workers is divided into three steps:

Step 1: Parallel detection and segmentation of human co-workers in RGB and thermal images.
Step 2: Determining the corresponding 2D heatmaps in the robot coordinate system R.
Step 3: Fusing of the resulting position information based on the classification confidence levels.

Object detection in image data has recently made remarkable progress through techniques from the field of machine learning. Deep neural networks are robust against a high variance in the object appearance compared to classical image processing methods. Corresponding models can detect or even segment objects under different environmental conditions, which makes them feasible for a human co-worker detection.

The detection process is performed redundantly. The RGB images $RGB_{i}\left\{i=1,2,3\right\}$ and the thermal image $T_{j}\left\{j=1,2,3,4,5,6\right\}$, are analyzed by object detection and segmentation algorithms in parallel. The YOLOv3 [52] model is employed for the objection detection task, which provides a bounding box and a corresponding confidence level ${\hat{\alpha }}_{B}$ for each object. For analyzing the RGB image data, we used public weights trained on the COCO dataset [53]. For the thermal images the model is trained with the Flir ADAS dataset [54]. Afterwards we fine-tuned the model using 1000 additional thermal images captured with the Flir Lepton. The images were taken at three different locations: an exhibition, an office and a terrace. We annotated the images at pixel level.

**Figure 7:**
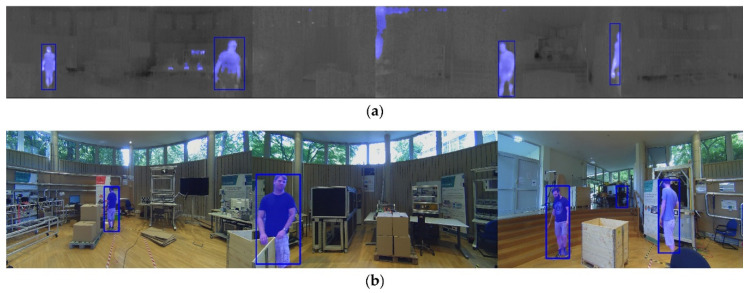
Human co-worker detection. (**a**) Thermal images $T_{j}$; (**b**) RGB images $RGB_{i}$.

The object segmentation is based on [55], which provides the confidence level ${\hat{\alpha }}_{xy}$ at pixel level. For analyzing the RGB image data, we used public weights available in literature [56]. For the thermal images we trained the model with the dataset presented in [57] and performed a fine-tuning step with the self-annotated dataset. Figure 7 shows one exemplary detection set of the WMS at a confidence level of ${\hat{\alpha }}_{B}>0.2$ and ${\hat{\alpha }}_{xy}>0.2$. The detected objects are represented by bounding boxes and the semantic segmentation is represented by blue blend masks.

The two sensor data types and the two detection methods result in four detectors labelled as experts E as follows:

- $E_{Y-RGB}:$ Bounding-box-based detection of the images $RGB_{i}$.
- $E_{Y-Thermal}:$ Bounding-box-based detection of the images $T_{j}$.
- $E_{B-RGB}:$ Segmentation-based detection of the images $RGB_{i}$.
- $E_{B-Thermal}:$ Segmentation-based detection of the images $T_{j}$.

**Figure 8:**
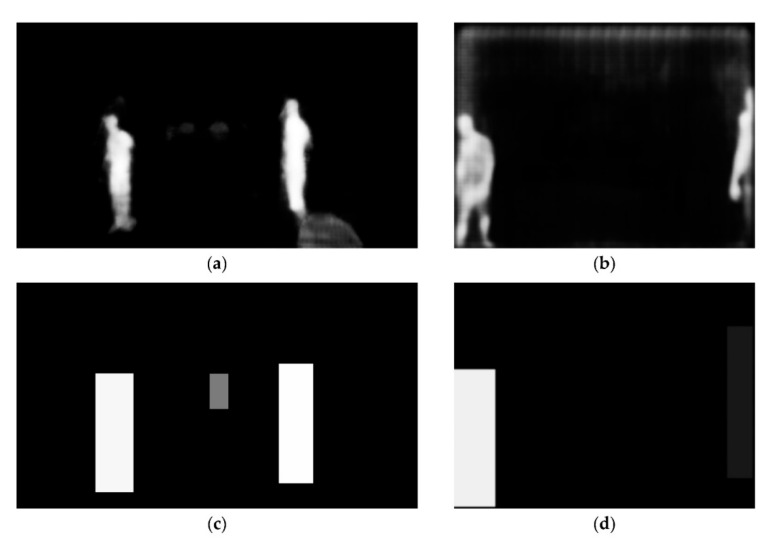
Gray scale image masks. (**a**) $RGB_{3}$ and expert $E_{B-RGB}$; (**b**) $T_{5}$ and expert $E_{B-Thermal}$; (**c**) $RGB_{3}$ and expert $E_{Y-RGB}$; (**d**) $T_{5}$ and expert $E_{Y-Thermal}$.

Each expert computes 2D image masks reflecting the detection and segmentation results. The image masks contain the classification results at pixel level, which are represented by the confidence levels ${\hat{\alpha }}_{B}$ for the experts $E_{Y-RGB}$ and $E_{Y-Thermal}$ as well as the confidence levels ${\hat{\alpha }}_{xy}$ for the experts $E_{B-RGB}$ and $E_{B-Thermal}$. Figure 8 shows the image masks of the images $RGB_{3}$ and $T_{5}$ of the detection set demonstrated in Figure 7. The confidence levels are normalized to a range of 0–255 in the gray scale masks for visualization purposes.

**Figure 9:**
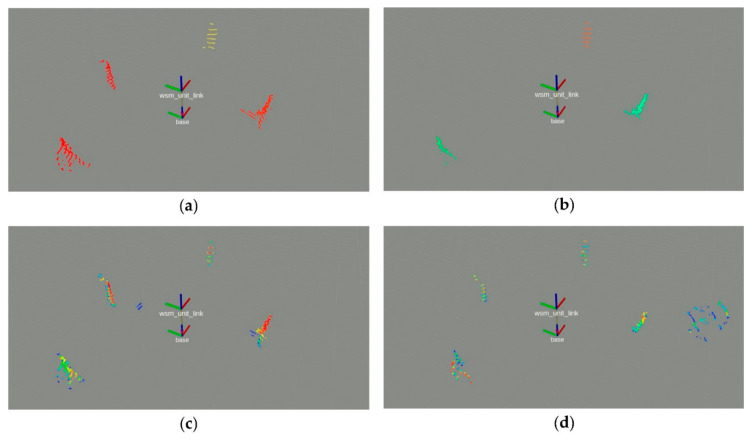
Point clouds representing the presence of a human co-worker. (**a**) $E_{Y-RGB}$; (**b**) $E_{Y-Thermal}$; (**c**) $E_{B-RGB}$; (**d**) $E_{B-Thermal}$.

In the next step, each expert projects the 3D points of the point cloud C into the image plane of the image masks. An a priori performed calibration provides the underlying projection matrix. The approach is presented in [58]. The expert extracts a subset of 3D points S⊂C based on the pixel coordinates of each image mask and the related lidar-camera projection matrix. Each 3D point is assigned the confidence level ${\hat{\alpha }}_{B}$ or ${\hat{\alpha }}_{xy}$, depending on the expert type. The resulting subset S is clustered using the Euclidean point-to-point distances to remove outliers. Figure 9 shows the final subsets $S_{H}\subset S$ for each individual expert of the detection set demonstrated in Figure 7. Each 3D point is colored according to the related confidence level. Red indicates a confidence level of 1 and blue a confidence level of 0.

The 3D points of the final subset $S_{H}\subset S$ are projected into a 2D grid pattern with the size of 12 m by 12 m and each cell sized 0.2 m by 0.2 m. The origin is located in the center of the grid pattern, corresponding to the robot coordinate system R. The experts use an "argmax" function over all points associated to one cell to calculate the confidence level $\hat{\beta }$ of each cell. An additional fifth expert $E_{Fusion}$ performs the confidence fusion. The fused confidence level $\hat{\gamma }$ is calculated for each cell by applying the logarithmic opinion pool [59] shown in Equation (1).

(1) $$\hat{\gamma }=\;\frac{\prod_{i=1}^{N}{\hat{\beta }}_{i}{}^{1/N}}{\prod_{i=1}^{N}{\hat{\beta }}_{i}{}^{1/N}+\;\prod_{i=1}^{N}{\left(1-{\hat{\beta }}_{i}\right)}^{1/N}}$$

**Figure 10:**
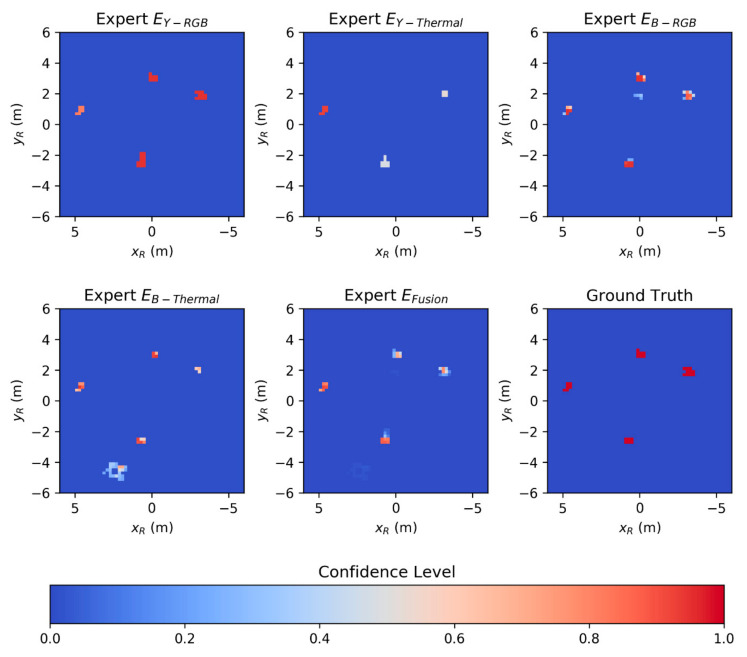
Heatmaps generated by the five different experts of the workspace monitoring system (WMS) and the ground truth.

The different confidence levels ${\hat{\beta }}_{i}$ are weighted equally, with N=4 representing the four experts. Equation (1) will result in $\hat{\gamma }=1$ if any of the confidence levels ${\hat{\beta }}_{i}$ is equal to 1. In another way, if any confidence level is equal to 0, then $\hat{\gamma }=0$. This "veto" behavior [60] is avoided by replacing $\hat{\beta }=0$ with $\hat{\beta }=0.01$ and $\hat{\beta }=1.0$ with $\hat{\beta }=0.99$, respectively [61]. Figure 10 shows the different heatmaps provided by the individual experts and the related ground truth.

### 3.4. Autonomous Navigation

The developed navigation concept is inspired by the zone management within an industrial production environment. The approach is based on virtual navigation zones, which can be defined using 2D polygons. The intuitive concept reduces the complexity of implementing AIMMs in industrial production environments. The virtual zones can be easily adapted to changes in the production layout and do not require any infrastructural modifications. The approach is scalable to numerous zones, including static and dynamic zone types. The zones are used to switch between predefined behaviors of the mobile platform with different settings, such as maximum speed and acceleration, the underlying kinematic model, warning indicators (visual and acoustic), the minimum distance to obstacles and the goal tolerance. It is also possible to switch between different path planners.

**Figure 11:**
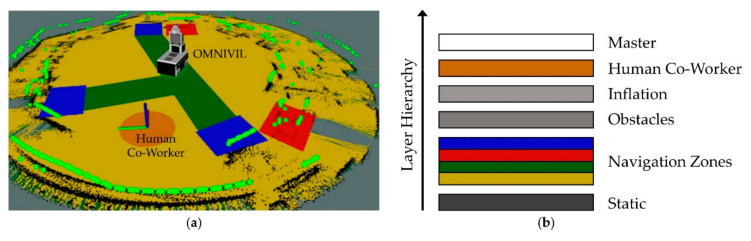
Navigation concept. (**a**) Zone setup without inflation layers; (**b**) costmap layers.

For instance, Figure 11a shows an example navigation zone setup in the model factory. The green zones are preferred zones for transportation. The mobile robot can enter the yellow zones if no path through the green zone is available. Entering the yellow zones will result in a decrease of the maximum velocity and a visual warning. The blue zones are goal zones related to individual workstations. Therefore, in the blue zones the maximum speed is reduced significantly and the goal tolerance is decreased to achieve a high positioning accuracy. The red zones are forbidden zones, which are in front of manual workbenches in the exemplary scenario. All the four zone types are static. In contrast, the orange zone is a dynamic zone representing a human co-worker. The creation of the orange zones is based on the heatmap H, representing the 2D positions of human co-workers detected by the WMS. Entering an orange zone will result in a decrease of the velocity, a warning and an increase of the minimal distance to obstacles. The path planning task belonging to the virtual zones is implemented by a layered costmap configuration [62]. The ordering of the layers allows modulating the costs by overwriting them only when and where required. Figure 11b shows the layers in the global costmap with the data being evaluated from bottom to top.

The virtual zone concept relies on accurate localization, obtained by using extended Kalman filtering [63,64]. The localization is split into local and global coordinate systems. The local localization fuses the linear velocities ${\dot{x}}_{O},{\dot{y}}_{O}$ and the angular velocity ${\dot{\theta }}_{O}$, provided by the platform odometry, with the angular velocity ${\dot{\theta }}_{I}$ provided by the IMU. The global localization is based on the ROS module 2D Cartographer [65], which uses a "Ceres"-based [66] scan matcher. Therefore, the laser scan data provided by the two 2D Lidars are merged to cover a 360° FOV around the robot origin. The global localization is based on a prior generated grid map of the production environment and provides a global pose $P_{W}=\;{\left(x_{W},\;y_{W},\;\theta_{W}\right)}^{T}$ in reference to the static world coordinate system W.

An intermediate path planner enables the adaption of existing path planning algorithms, e.g., A* [67] or Dijkstra [68], to the proposed navigation zone concept. The Radish planner splits the global path from start to goal pose into smaller sub-paths, according to individual zones. The Radish planner aims to provide intermediate waypoints so that the robot footprint stays in preferred zones for as much of the trajectory as possible. The zones are ordered according to the fixed costs of the individual costmap. For the zone setup of the model factory in Figure 11, two intermediate waypoints are necessary, which are in the preferred green transport zone. The first intermediate waypoint is related to the robot start pose $P_{s}=\;{\left(x_{s},\;y_{s},\;\theta_{s}\right)}^{T}$. The second intermediate waypoint is related to the desired goal pose $P_{g}=\;{\left(x_{g},\;y_{g},\;\theta_{g}\right)}^{T}.$ An intermediate waypoint is considered valid if at least 80% of the robot’s footprint is in the green zone. The radish planner aims to find the closest valid waypoint based on an initial pose $P_{i}=\;{\left(x_{i},\;y_{i},\;\theta_{i}\right)}^{T}$. Starting from the robot pose $P_{i}$ a circular search is performed with an angular sampling rate $\Delta \theta_{1}$, according to the Equations (2) and (3). The sampling rate $\Delta \theta_{1}$ determines the number of n positions ${\left(x_{i+n},\;y_{i+n}\right)}^{T}$ alongside the circle with the radius r.

(2) $$x_{i+n}=x_{i}+r*\cos\left(\Delta \theta_{1}*n\right)$$

(3) $$y_{i+n}=y_{i}+r*\sin\left(\Delta \theta_{1}*n\right)$$

The robot footprint is non-circular, therefore the initial orientation angle $\theta_{i}$ is variated at each position with a sampling rate of $\Delta \theta_{2}$, according to Equation (4). The sampling rate $\Delta \theta_{2}$ determines the number of m intermediate waypoints ${\left(x_{i+n},\;y_{i+n},\;\theta_{i+m}\right)}^{T}$ at each position ${\left(x_{i+n},\;y_{i+n}\right)}^{T}$.

(4) $$\theta_{i+m}=\theta_{i}+\Delta \theta_{2}*m$$

If none of the calculated intermediate waypoints is feasible, the search radius r is increased and the process is repeated. The procedure is cancelled if the radius r is greater than a defined threshold. In that case no intermediate waypoint related to the pose $P_{i}$ is used for the path planning task. This procedure is executed for both, the start and goal pose of the robot.

### 3.5. Adaptive Manipulation

The 6D motion planning for the industrial manipulator is performed using the ROS module MoveIt [69], which provides sensor data integration for workspace monitoring and monitors the state of the manipulator via its joint positions. In addition, it features different state-of-the-art motion planners including the Open Motion Planning Library (OMPL [70]).

Industrial manipulators provide a high repeatability, which is important for repetitive motion execution. However, repetitive motion execution requires a deterministic environment and a well-defined task description. In contrast to static manipulators, a mobile manipulator is a dynamic system. The positioning of the mobile manipulator will vary each process step, caused by small navigation errors or ground unevenness. An adaptive manipulation must compensate small positioning errors of the mobile platform each time to provide stable task execution.

**Figure 12:**
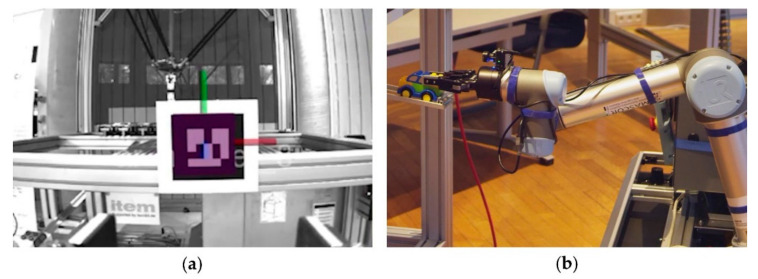
Grasping task based on visual servoing. (**a**) Augmented Reality (AR)-marker detection and 3D pose estimation; (**b**) grasping process of a Lego car.

Therefore, a visual servoing approach based on Augmented Reality (AR)-marker is used to adapt the 6D motion planning. The AR-markers are mounted at the different workstations. The 6D grasp poses are known in the coordinate systems of the AR-markers A. The 6D pose of an AR-marker $P_{ar}={\left(x_{ar},\;y_{ar},\;z_{ar},\;roll_{ar},\;pitch_{ar},\;yaw_{ar}\right)}^{T}$ in the robot coordinate system R is calculated based on image data provided by the RGB-D camera using the open-source AR-marker tracking library ALVAR [70]. Figure 12 shows the detection of the AR-marker and the grasping task performed by the mobile manipulator. For the initial approach OMPL’s implementation of RRT-Connect [71] has proven itself to be reliable. MoveIt’s cartesian path planning capability is used to calculate the final linear approach and escape paths.

**Figure 13:**
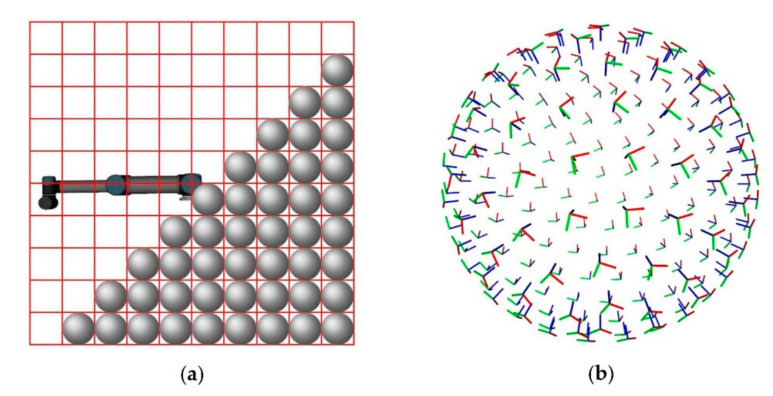
Workspace analysis. (**a**) Schematic of the workspace divided into a voxel grid; (**b**) spherically arranged set of 6D poses $P_{ee}$ for one voxel.

The workspace of a manipulator is inhomogeneous in terms of reachability and manipulability. The reachability describes the capability of the end-effector to reach a 6D pose $P_{ee}={\left(x_{ee},\;y_{ee},\;z_{ee},\;roll_{ee},\;pitch_{ee},\;yaw_{ee}\right)}^{T}.$ The manipulability is the capability to move the end-effector in a specific direction given a 6D pose $P_{ee}$. Therefore, a limiting factor for the manipulation process is the positioning of the mobile platform in relation to the desired end-effector pose. The determination of an optimal position of the mobile platform can be time consuming and requires expertise in robotics. A simple adaption to different tasks is a core functionality of a flexible mobile manipulator in an industrial environment. Therefore, the adaptive manipulation module of the mobile manipulator OMNIVIL includes a workspace analyzing tool to reduce the complexity. The tool is based on the core idea presented in [72]. It provides a visualization for reachability tasks, e.g., pick and place, as well as manipulability tasks, such as polishing or inspection. Therefore, the workspace of the manipulator is discretized to a 3D voxel grid as shown in Figure 13a. We used the algorithm presented in [73] to create the spherical arranged set of 6D end-effector poses for each voxel. Figure 13b shows the equally distributed end-effector poses.

A 6D pose $P_{ee}$ is reachable if the inverse kinematic of the manipulator is solvable. The reachability index d [72] of an individual voxel is described in Equation (5),

(5) $$d=\;\frac{a}{b}*100,$$

where a is the number of reachable end-effector poses and b is the number of all end-effector poses in the voxel.

At each reachable end-effector pose the manipulability index w [74] is calculated following Equation (6),

(6) $$w=\sqrt{\det\left(J_{ee}*J_{ee}{}^{T}\right)},$$

where $J_{ee}$ is the Jacobian matrix for the end-effector at that robot configuration and $J_{ee}{}^{T}$ its transpose. The calculation of the Jacobian is given in Equation (7),

(7) $$J_{ee}\left(q\right)=\frac{\partial P}{\partial q}=\;\left[\begin{array}{ccc} \begin{array}{cc} \begin{array}{c} \frac{\partial P_{1}}{\partial q_{1}}\\ \frac{\partial P_{2}}{\partial q_{1}} \end{array} & \begin{array}{c} \frac{\partial P_{1}}{\partial q_{2}}\\ \frac{\partial P_{2}}{\partial q_{2}} \end{array} \end{array} & \cdots & \begin{array}{c} \frac{\partial P_{1}}{\partial q_{6}}\\ \frac{\partial P_{2}}{\partial q_{6}} \end{array}\\ \vdots & \ddots & \vdots \\ \begin{array}{cc} \frac{\partial P_{6}}{\partial q_{1}} & \frac{\partial P_{6}}{\partial q_{2}} \end{array} & \cdots & \frac{\partial P_{6}}{\partial q_{6}} \end{array}\right],$$

where P is the vector of the end-effector pose and q the vector representing the joint angle configuration. The manipulability index provides an indication of how well a pose $P_{ee}$ can be adjusted at a given robot configuration. A voxel is assigned the average of the manipulability indices $\bar{w}$ of all reachable poses $P_{ee}$. Voxels with no reachable poses $P_{ee}$ are assigned a manipulability index of 0.

**Figure 14:**
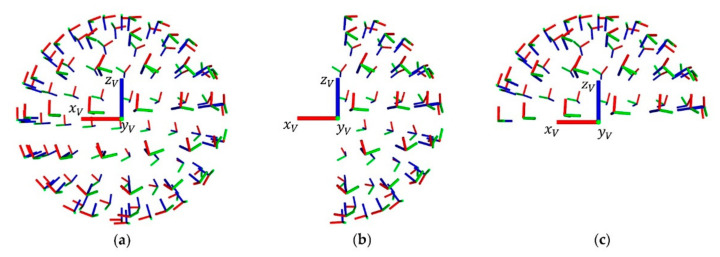
Sets of 6D end-effector poses to calculate reachability and maneuverability. (**a**) full sphere; (**b**) hemisphere pointing in forward x-direction; (**c**) hemisphere pointing in downward z-direction.

An analysis of the workspace based on spherically oriented poses $P_{ee}$ provides a good indication of the workspace in terms of reachability and manipulability. However, in many industrial applications only subsets of these end-effector poses are of interest. Typical examples are pick and place applications, which require a downward facing end-effector or inspection applications, which require a forward-facing end-effector. Therefore, the spherical approach is extended by two additional hemispheres. One hemisphere includes end-effector poses which are oriented to the front and the other hemisphere includes end-effector poses oriented to the ground in respect to the robot coordinate system R. Figure 14 shows a comparison of the three geometrical distributions. The voxel coordinate system V is equally oriented for each voxel.

### 3.6. Integration in a Model Factory

**Figure 15:**
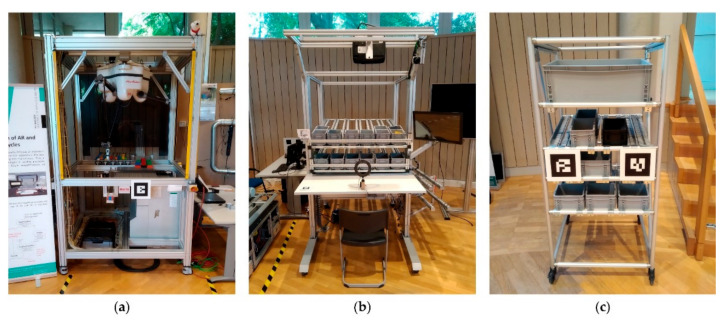
Workstations in model factory to be approached by the mobile manipulator. (**a**) Robot cell with delta picker; (**b**) manual workbench with augmented reality support; (**c**) ware-rack.

The developed mobile manipulator OMNIVIL is used in a model factory [75] for experimental purposes. One example product of the model factory is a Lego car (see Figure 12), which is produced according to the customers’ requirements (color and model). The production process includes several workstations: a fully automated robot cell equipped with an industrial delta picker, manual workbenches with AR support and a warehouse system. In an exemplary use case, OMNIVIL transports goods and preassembled parts between the workstations. Figure 15 shows the three workstations, which are approached by OMNIVIL.

## 4. Experiments and Discussion

### 4.1. Localization and Positioning Accuracy

**Figure 16:**
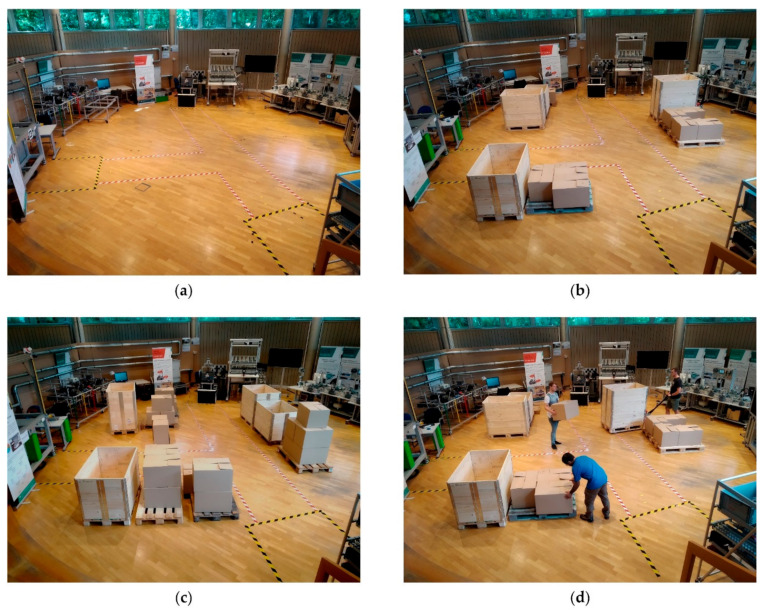
Localization test scenarios. (**a**) $S_{static}$; (**b**) $S_{crowded}$; (**c**) $S_{crowded*}$; (**d**) $S_{dynamic}$.

We tested the mobile manipulator OMNIVIL in an exemplary logistic use case, implemented in the model factory. The localization experiment includes four different scenarios S, reflecting typical variations in industrial environments. The scenario changes in the experiments are located between 0 und 2 m height, comparable to an industrial production scenario:

- $S_{static}$: No changes compared to the prior generated map (see Figure 16a).
- $S_{crowded}$: Minor static changes compared to the prior generated map (see Figure 16b).
- $S_{crowded*}$: Many static changes compared to the prior generated map (see Figure 16c).
- $S_{dynamic}$: This scenario is based on $S_{crowded}$ but includes dynamic changes. Three human co-workers are continuously transporting boxes and pallets with help of a manual lift truck (see Figure 16d).

**Figure 17:**
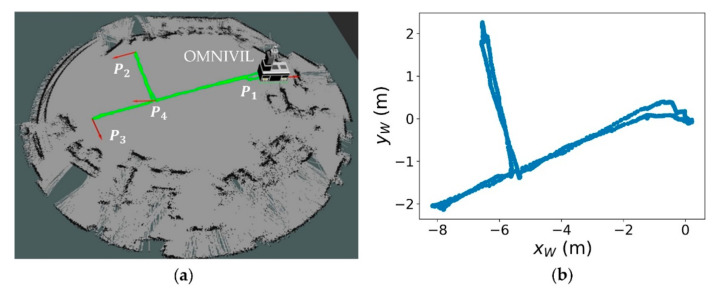
Sequential movement through the model factory. (**a**) Executed path and positions $P_{1}$, $P_{2}$, $P_{3}$ and $P_{4}$, with OMNIVIL parking at reference pose $P_{1}$; (**b**) executed path in the static world coordinate system W.

The sensing data was stored during 40 runs in each scenario and postprocessed offline, which creates a comparable database. As depicted in Figure 17a, each run includes a full movement of the mobile manipulator between the three workstations in the model factory, whereby the robot was controlled manually.

Figure 17b shows one performed movement process starting from pose $P_{1}$ and moving to pose $P_{2}$ and $P_{3}$ sequentially, before returning to $P_{1}$. The position $P_{1}$ is set as reference position to estimate the localization errors and standard deviations. The final positioning of the platform at the position $P_{1}$ is secured by mechanical end stops. One complete run has an absolute Euclidean path length of approximately 33 m. It includes several rotations, which sum up to an absolute rotation of around 12.5 rad (about two revolutions).

The differences between the scenarios are compared by the average range value changes in a period of 2D laser beam scanning 360°. This factor of change A is calculated following Equation (8).

(8) $$A=\frac{\sum_{i=0}^{n_{l}}\frac{\left|M_{i}-R_{i}\right|}{R_{i}}}{n_{l}},$$

where $M_{i}$ is the range value of the laser beam i in the current environmental scenario and $R_{i}$ is the range value of the laser beam i in the reference environmental scenario $S_{static}$. The constant $n_{l}$ is the number of valid beams per laser scan. The factor of change A is determined at position $P_{4}$ (see Figure 17. Table 2 shows the factor of change for the three static scenarios.

The localization experiment compares four different localization strategies feasible with the implemented sensor concept. All localization approaches use a prior created map. Such pure re-localization strategies minimize the necessary computing power compared to simultaneous localization and mapping (SLAM) approaches.

**Figure 18:**
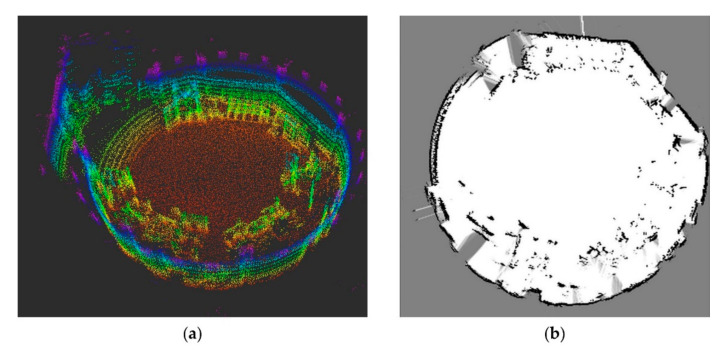
A priori maps used for localization experiments. (**a**) 3D point cloud map without roof; (**b**) 2D occupancy grid map.

The 2D map is created in form of an occupancy grid map using OpenSlam’s gmapping [76], which is based on Rao-Blackwellized particle filters, described in [77,78]. The sparse 3D map is created using the 3D SLAM Algorithm LeGO-LOAM [79]. Figure 18 shows the resulting 3D point cloud and the 2D occupancy grid map.

The localization is divided into global and local localization. The local localization is based on the platforms odometry and the IMU data. The local localization provides a mean absolute error of 0.0734 m ± 0.023 m in *x*-direction, 0.253 m ± 0.067 m in *y*-direction and 0.008 rad ± 0.007 rad for *θ*. The calculation is given in Equation (9),

(9) $$MEA_{local}=\;\frac{\sum_{i=1}^{n}\left|P_{i}-P_{i+1}\right|}{n-1},$$

where n is equal to the total amount of 160 runs and $P_{i}\left\{i=1,\ldots ,n\right\}$ is the output of the resulting local localization at position $P_{1}$ for run i. The relatively small systematic error in combination with the low standard deviation σ qualifies the implemented local localization approach to be used in combination with the global localization approaches.

The global localization uses fixed features according to a static global frame. Four different global localization approaches, $L_{i}\left\{i=1,2,3,4\right\}$, are compared using 2D or 3D Lidar data:

- $L_{1}:$ 2D Adaptive Monte Carlo localization (AMCL) [80].
- $L_{2}:$ 2D CARTOGRAPER [65], which uses a Ceres-based [66] scan matcher.
- $L_{3}:$ 3D Monte Carlo localization [81], which is based on the Lidar measurement models described in [82] and makes use of an expansion resetting method [83].
- $L_{4}:$ 3D HDL Localization [84], which iteratively applies normal distributions transform (NDT) scan matching [85].

**Figure 19:**
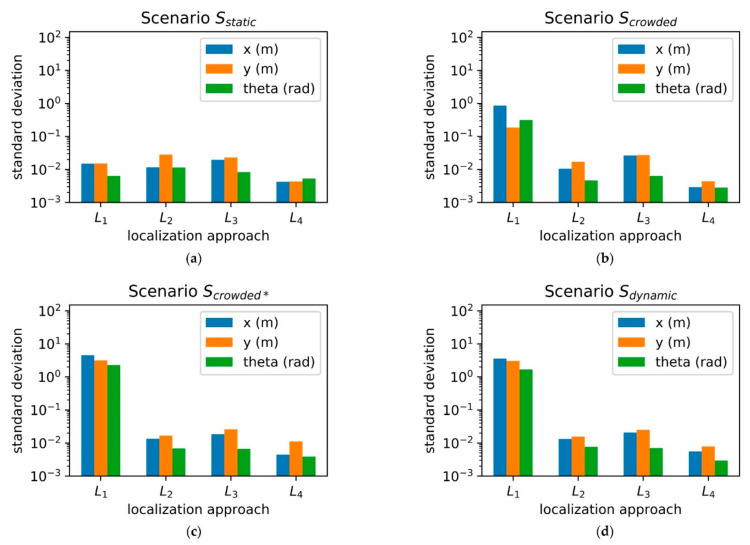
Localization experiment results. (**a**)$\;S_{static}$; (**b**) $S_{crowded}$; (**c**) $S_{crowded*}$; (**d**) $S_{dynamic}$.

The conducted experiment compares the global localization approaches in four different scenarios. The sample size is 40 runs for each scenario. In contrast to the local localization, an estimation of the systematic error is not possible, as it would require a highly precise 6D reference system. However, the standard deviation measured at the reference position $P_{1}$ gives a good indication of the performance. Figure 19 shows the experimentally determined results.

Except for $L_{1}$, all global localization approaches provide a high robustness against static and dynamic changes in the environment, i.e., small standard deviation values. The 3D localization strategies $L_{3}$ and $L_{4}$ make use of features, which are not affected by the static and dynamic changes, because they are located on a higher spatial level. The algorithm used in $L_{2}$ shows a comparable reliability, proving that a 2D localization can be used in such scenarios as well. The best performance was provided by $L_{4}$.

Another important factor besides accuracy is the computational load of the different methods. We performed the evaluation offline using a 12 core Intel i7-8700 @3.2GHz with 32 GB RAM. Table 3 shows a comparison of the computational load and the memory usage for each global localization strategy.

We conducted an additional experiment to evaluate the positioning accuracy of the mobile platform during autonomous navigation. We choose the best performing localization approach $L_{4}$ and compared it with the best 2D localization approach $L_{2}$. In addition, we evaluated a marker-based positioning approach. Therefore, we equipped each workstation with an additional AR-Marker. The forward-facing camera of the mobile platform detects the marker. The pose of the marker is determined using the open-source AR-marker tracking library ALVAR. The platform approaches reactively the desired goal pose in relation to the marker using a PID controller.

The experimental procedure to evaluate the positioning error is based on a camera-marker setup. The setup of the reference system is similar to [86]. The starting pose for the autonomous navigation task was set to the initial pose $P_{4}$. The goal poses are the three workstation poses $P_{1}$, $P_{2}$, and $P_{3}$. Each goal pose was approached 40 times in the scenario $S_{static}$. The goal tolerance of the local planner was set to 5 mm in *x*- and *y*-direction and 0.005 rad for *θ*. Table 4 shows the standard deviation σ of the positioning accuracies at the different goal poses for the evaluated positioning strategies.

The marker-less localization strategies $L_{2}$ and $L_{4}$ proved, that they are comparable to a marker-based positioning of the mobile manipulator. The approach based on $L_{4}$ even provides a superior positioning accuracy compared to the marker-based approach. The conducted experiments show the positioning capabilities of the autonomous mobile manipulator OMNIVIL and proved that a robust and precise localization and path execution is possible without any infrastructural modifications in the production environment.

### 4.2. Human Co-Worker Detection

**Figure 20:**
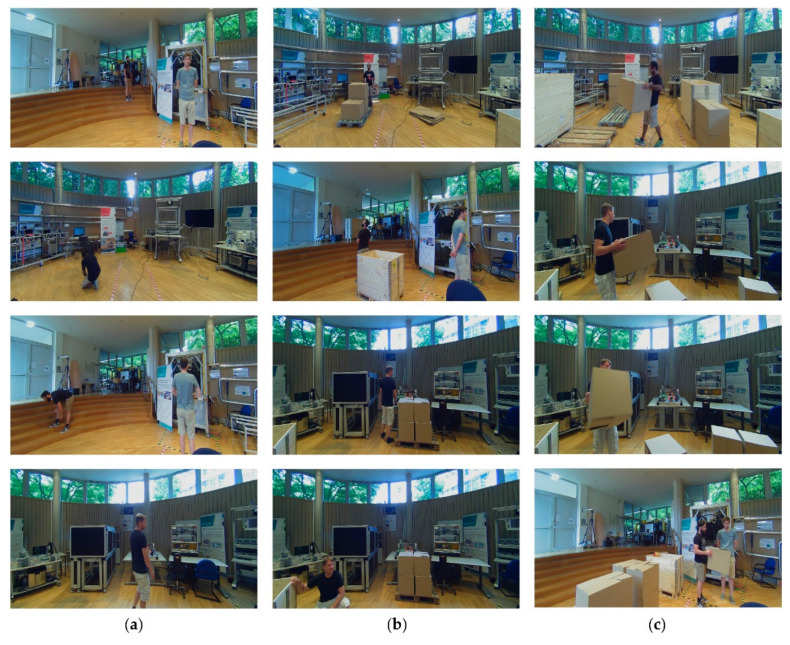
Exemplary dataset subsegments. (**a**)$\;S_{static}$; (**b**) $S_{crowded}$; (**c**) $S_{dynamic}$.

We performed an experiment to validate the performance of the presented WMS system. Therefore three different datasets were created in the three scenarios $S_{static}$, $S_{crowded}$, and $S_{dynamic}$. In contrast to the localization experiments up to four human co-workers are present in the scenarios. Each dataset consists of 190 dataset subsegments, which sums up to 570 subsegments in total, including various edge cases. Figure 20 gives an exemplary overview of the datasets.

For each dataset, the ground truth subsegments for the human co-workers were manually annotated. To evaluate the performance of the five experts, the resulting confidence heatmaps are analyzed as following: Let N be a threshold of the confidence level, $C_{xy}$ a cell of the expert heatmap and $G_{xy}$ the corresponding cell of the ground truth heatmap. Then $C_{xy}$ is interpreted as a positive detection $C_{xy}^{+}$ if the confidence level L > N. Otherwise it is not considered. All positive detections are divided into true positive and false positive detections as follows:

(10) $$C_{xy}^{+}=\;\left\{\begin{array}{cc} \;true\;positive,\; & G_{x,y}+G_{x+1,y}+G_{x-1,y}+G_{x,y+1}+G_{x,y-1}\geq 1\\ false\;positive, & else \end{array}\right.$$

**Figure 21:**
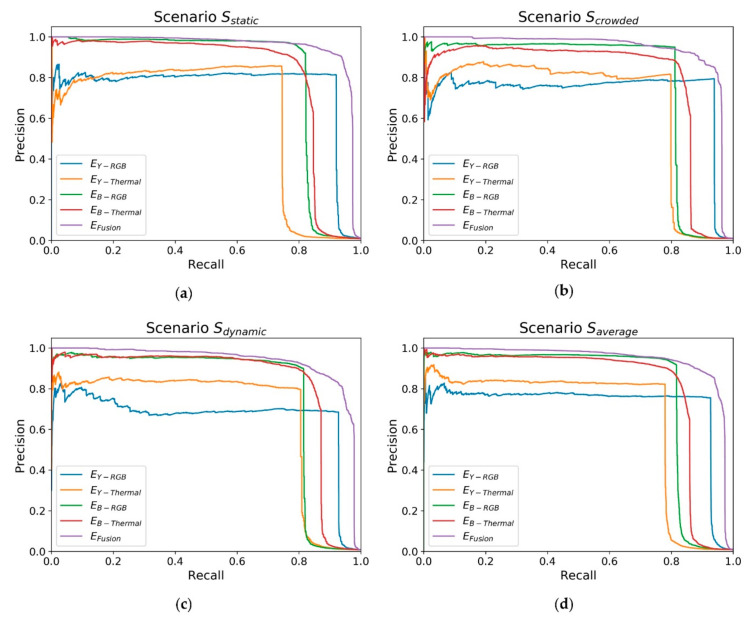
Evaluation of the expert performances in different scenarios. (**a**) $S_{static}$; (**b**) $S_{crowded}$; (**c**) $S_{dynamic}$; (**d**) all scenarios ($S_{average}$).

A positive detection $C_{xy}^{+}$ is scored as a true positive if the corresponding ground truth $G_{xy}$ or at least one of its close neighbored cells is marked as positive (equals to one). A similar procedure is used to divide false detections $C_{xy}^{-}$ into false negative and true negative detections. A negative detection $C_{xy}^{-}$ is scored as false negative if the corresponding ground truth $G_{xy}$ or at least one of its close neighbored cells is marked as positive. Figure 21 shows a comparison of the various expert performances based on their characteristic precision/recall curve.

The average precision (AP) is calculated for each expert as shown in Equation (11),

(11) $$AP=\;\overset{n}{\underset{i=1}{\sum }}\left(R_{i}-R_{i-1}\right)*P_{i},$$

where n is the number of measurements, $R_{i}$ is the recall and $P_{i}$ is the precision value at the measurement i.

Table 5 shows the AP values in comparison.

The reliability is the basic requirement for a safety module like the presented WMS system. Both precision and recall are equally important. A low precision will result in more false positive detections, obstructing the zone-based navigation. A lower recall indicates more missed human co-workers, which will deny the desired preventive behavior. The experts based on semantic segmentation provide a higher AP compared to the experts based on object detection. This is caused by the related image masks. The image masks based on the object detection approach contain rectangular boxes around the detected objects, whereby the image masks based on semantic segmentation are reflecting the contour of the object.

Apart from the expert $E_{Fusion}$, the expert $E_{B-Thermal}$ achieved the best individual score with an AP of 0.83 in the dynamic scenario. The expert $E_{B-Thermal}$ shows comparable performance to its RGB counterpart. Both results support the hypothesis that thermal data is well suited to determine human co-workers in cluttered environments.

The AP of the four basic experts fluctuates between 0.62 and 0.83 in the four different scenarios, which is not suitable for a safety system. In contrast, the expert $E_{Fusion}$ shows a fluctuation of only 0.02 over the four different scenarios. Its performance surpasses the best basic expert by over 10%. With an average precision of 0.94 over all three datasets the presented WMS system provides the reliability and precision to be used as a soft-safety system in a mobile manipulation application.

### 4.3. Workspace Analyzing

**Figure 22:**
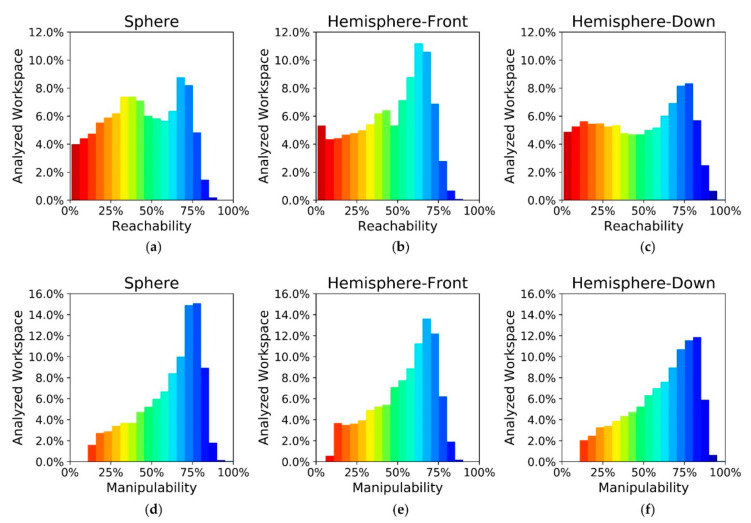
Workspace evaluation of the mobile manipulator OMNIVIL. (**a**) Reachability (spherical); (**b**) reachability (hemispherical-front); (**c**) reachability (hemispherical-down); (**d**) manipulability (spherical); (**e**) manipulability (hemispherical-front); (**f**) manipulability (hemispherical-down).

**Figure 23:**
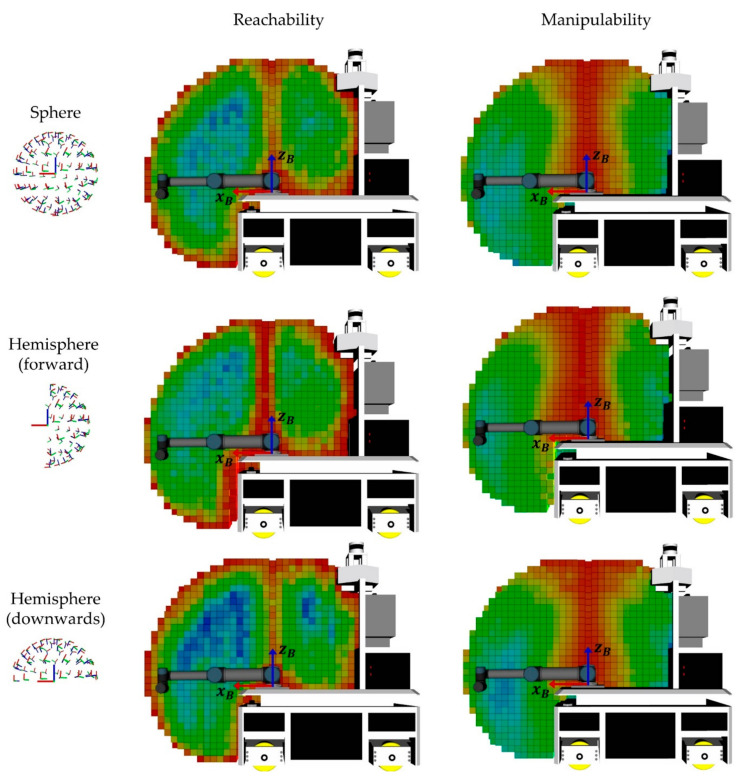
Workspace analyzing divided into reachability and manipulability.

The workspace evaluation of the mobile manipulator OMNIVIL is based on a robot model to identify self-collisions. The reachability is determined by analyzing 100 evenly distributed end-effector poses for three geometric distributions, namely: sphere, hemisphere-front and hemisphere-down (see Section 3.5). Figure 22 shows the results of the workspace evaluation. For reasons of simplification, we neglected voxels which are located at negative x-positions in the base coordinate system B of the manipulator (see Figure 23). The scalar values for the manipulability indices were normalized to 0–100% for each distribution.

The reachability values depend on the geometric distribution of the end-effector poses. In case of a forward-facing hemisphere, the number of voxels providing a reachability of 50–75% is increased by 9%, compared to the spherical distribution. The downward-facing hemisphere results in more voxels with a reachability over 75%. Compared to the spherical distribution, the number is increased by 11%. Both results support the hypothesis that it is important to determine a geometrical distribution that matches the actual application.

In contrast to reachability, the manipulability index does not provide absolute values. However, the manipulability indices provide a good indication, which areas in the workspace of the manipulator should be preferred for manipulation tasks. Similar to the reachability, the values of the manipulability depend on the geometric distribution of the end-effector poses.

We developed a visualization tool to demonstrate the reachability and manipulability in the workspace of the manipulator. The tool includes functionalities such as adjustable 3D filters and different transparency levels. Therefore, the proposed visualization provides an intuitive way for the operator to position the platform in a spot, which offers high reachability or manipulability capabilities. Figure 23 shows a vertical visualization of the OMNIVIL robot model and the workspace visualization.

### 4.4. Comparsion with Existing Mobile Manipulators

In the last decade various mobile manipulators were developed in research. Most of them are based on a commercially available mobile platform. Nowadays, even complete mobile manipulators are available on the market. Table 6 compares some of the most recent industrial mobile manipulators.

All mentioned mobile manipulators are equipped with 2D safety Lidars as a hard-safety component, which is common for autonomous vehicles. Advanced sensor technology or even machine learning methods can achieve a high level of soft-safety to enable intuitive HRC. Therefore, the researched mobile manipulators are equipped with various sensors for obstacle detection, namely: RGB-D cameras, ToF cameras, stereo-cameras, and light-field cameras. In addition, all mobile manipulators are equipped with a collaborative manipulator, to enable true HRC. Another promising approach in this direction is the implementation of artificial skins, which can detect proximity or contact forces as well as the related impact location. In contrast to industrial grade hard-safety components, these soft-safety concepts are error-prone and difficult to maintain. Redundancy and the combination of different technologies is crucial to overcome these problems [96]. Therefore, OMNIVIL makes use of a multilayer sensor concept and a redundant data analysis. OMNIVIL is the only mobile manipulator of the evaluated systems, which classifies the detected obstacles.

The mobile manipulator Chimera is based on the commercial platform MiR100 [97]. The MiR100 platform provides a navigation based on static zones. OMNIVIL enlarges this concept by the implementation of dynamic zones. These dynamic zones represent the position of human co-workers in the environment. As a result, OMNIVIL is the only system, which provides preventive behavior adaption in the context of HRC.

## 5. Conclusions

This study presented the development and implementation of the autonomous mobile manipulator OMNIVIL. It provides insights in the related research fields including autonomous navigation, visual-servoing, workspace monitoring, and 6D motion planning.

The main goal of the development was the identification of technical issues, which discourage this technology from industrial applications and its acceptance. Safety concerns were addressed by combining hard-real-time safety components with a high-level soft-real-time workspace monitoring system. Experiments showed the superior redundant concept, proving the feasibility of human–robot collaboration in industrial use cases. The intuitive navigation zone concept, the adaptive visual-servoing approach and the workspace analyzing tool reduce the complexity of the mobile manipulator. An additional experiment evaluated the localization and positioning capabilities of the mobile platform, without the need of infrastructural modifications. The results show a high robustness against static and dynamic changes in the environment and a suitable accuracy for the execution of manipulation tasks.

Further improvements will focus on the workspace monitoring system. The scalable redundant concept will be extended by analyzing the Lidar data. Furthermore, the adaptive manipulation will be improved for marker-free scenarios to enable the proposed mobile manipulator OMNIVIL to be implemented in a production environment without any infrastructural needs.

## Figures and Tables

**Table 1:** Technical data of the mobile platform.

Description	Value
Dimensions	1256 × 780 × 522 mm³ (L × W × H)
Ground Clearance	42 mm
Weight	200 kg
Maximum Payload	380 kg
Maximum Velocity	1.3 m/s
Wheel Type	Mecanum
Kinematic	Holonomic

**Table 2:** Medium change per laser beam in the static scenarios relative to $S_{static}$.

Scenario	Valid Beams	Change Factor *A*
$S_{static}$	416	0.0
$S_{crowded}$	329	0.388
$S_{crowded*}$	388	0.512

**Table 3:** Computational load and memory usage of the global localization strategies.

Localization Strategy	CPU (% of Single Core)	RAM (GB)
$L_{1}$ (2D)	20	0.51
$L_{2}$ (2D)	46	3.14
$L_{3}$ (3D)	103	2.56
$L_{4}$ (3D)	240	1.92

**Table 4:** Positioning accuracy of autonomous navigation.

Localization Strategy	Goal Pose	σ_x_ (mm)	σ_y_ (mm)	σ*_θ_* (rad)
$L_{2}$	$P_{1}$ (workbench)	7	6	0.004
	$P_{2}$ (robot cell)	9	12	0.007
	$P_{3}$ (ware rack)	11	23	0.012
$L_{4}$	$P_{1}$ (workbench)	3	10	0.003
	$P_{2}$ (robot cell)	3	3	0.005
	$P_{3}$ (ware rack)	3	3	0.003
AR−Marker	$P_{1}$ (workbench)	9	5	0.01
	$P_{2}$ (robot cell)	5	10	0.01
	$P_{3}$ (ware rack)	10	12	0.02

**Table 5:** Comparison of the average precision.

	$S_{\mathrm{static}}$	$S_{\mathrm{crowded}}$	$S_{\mathrm{dynamic}}$	$S_{\mathrm{average}}$
**AP** $E_{Y-RGB}$	*0.75*	0.74	0.64	0.71
**AP** $E_{Y-Thermal}$	*0.62*	0.67	0.67	0.65
**AP** $E_{B-RGB}$	*0.81*	0.78	0.77	0.78
**AP** $E_{B-Thermal}$	*0.80*	0.79	0.83	0.81
**AP** $E_{Fusion}$	*0.95*	0.93	0.93	0.94

**Table 6:** Overview of related mobile manipulators developed in research and industry.

Institution/Company	Mobile Manipulator	Kinematic and Manipulator	Safety Features	Navigation Features
Aalborg University	LittleHelper [10]	differential KUKA LWR	2D safety Lidar, ultrasonic sensors	landmark-based localization at workstations
Joanneum Research	Chimera [87]	differential UR10	2D safety Lidar, RGB-D camera	static navigation zones
IPA Frauenhofer	Amadeus [13]	omnidirectional UR10	2D safety Lidar	localization based on induction wires
IPA Frauenhofer	rob@work [88]	omnidirectional configurable	2D safety Lidar	trajectory tracking along dynamic surfaces
IFF Frauenhofer	ANNIE [89]	omnidirectional KUKA LBR 4+	2D safety Lidars, a light-field camera, RGB camera	static landmarks to increase the localization accuracy
IFF Frauenhofer	VALERI [24,90,91]	Omnidirectional KUKA LWR	2D safety Lidars, bumper, stereo camera, Time of Flight (ToF) camera, tactile artificial robot skin	not focused
TUM	TOMM [92]	omnidirectional dual arm UR5	2D safety Lidars, RGB cameras, tactile artificial robot skin	not focused
Tecnalia	MRP [33]	omnidirectional dual arm UR10	2D safety Lidars, RGB-D, stereo-cameras	2D and 3D perception-based navigation
KUKA	KMR iiwa [93]	omnidirectional KUKA LBR iiwa	2D safety Lidars	+/− 5 mm positioning accuracy
KUKA	KMR QUANTEC [93]	omnidirectional KUKA KR Quantec	2D safety Lidars	+/− 5 mm positioning accuracy
Yaskawa	YMR12 [94]	differential MH12F, HC10	2D safety Lidars, 3D cameras, ToF camera	no information
Neobotix	MM-700 [95]	differential configurable	2D safety Lidars	no information
IaAM	OMNIVIL	omnidirectional UR5	2D safety Lidars, RGB-D camera, RGB cameras, thermal cameras, 3D Lidar	static and dynamic navigation zones
